# Terlipressin‐induced skin necrosis in cirrhotic patients—A case report and comprehensive literature review

**DOI:** 10.1002/ccr3.9141

**Published:** 2024-09-29

**Authors:** Ashraf I. Ahmed, Muhammad Zain Kaleem, Shahem Abbarh, Haider Hussein Barjas, Abdellatif Ismail, Mhd Kutaiba Albuni, Bisher Sawaf

**Affiliations:** ^1^ College of Medicine, QU Health, Qatar University Doha Qatar; ^2^ Department of Internal Medicine Hamad Medical Corporation Doha Qatar; ^3^ Department of Internal Medicine University of Maryland Medical Center‐Midtown Campus Baltimore Maryland USA; ^4^ Department of Internal Medicine Trihealth Good Samaritan Hospital Cincinnati Ohio USA

**Keywords:** esophageal variceal bleed, esophageal varices, hepatorenal syndrome, skin necrosis, terlipressin, upper gastrointestinal bleeding

## Abstract

**Key Clinical Message:**

The occurrence of terlipressin‐induced skin necrosis in cirrhotic patients is a rare but serious adverse event that warrants further investigation. Clinicians should be aware of this potential complication in cirrhotic patients receiving terlipressin therapy and closely monitor for any signs of skin necrosis. Early recognition and prompt intervention are crucial in preventing further complications and improving patient outcomes. Further research is needed to better understand the risk factors associated with terlipressin‐induced skin necrosis and to develop effective preventive strategies. Overall, healthcare providers should exercise caution when prescribing terlipressin to cirrhotic patients, weighing the potential benefits against the risks of this rare but significant adverse event.

**Abstract:**

Terlipressin is commonly used to manage conditions related to portal hypertension, such as hepatorenal syndrome and esophageal variceal bleeding. Despite its therapeutic benefits, terlipressin can rarely lead to severe ischemic complications involving the skin vasculature, known as terlipressin‐induced skin necrosis. We present a 50‐year‐old male with cirrhosis and acute variceal bleeding who developed skin necrosis following terlipressin administration. We performed a comprehensive review of the literature by analyzing 18 case reports/case series comprising 22 cirrhotic patients with terlipressin‐induced skin necrosis. Among these individuals, we found a mean age of 51 years with a male predominance (78%). Further analysis showed that the onset of skin necrosis ranged from 2 to 5 days post‐terlipressin initiation, with bolus administration being predominant (85.7%). The underlying pathophysiological mechanisms of terlipressin‐induced skin ischemia are still elusive but primarily attributed to the vasoconstrictive and thrombogenic effects. Management involves terlipressin discontinuation and supportive care. Physicians should be aware of this potential complication in patients receiving terlipressin and closely observe for any signs of skin rash.

## 1 INTRODUCTION

Cirrhosis is a chronic liver disease characterized by the replacement of healthy liver tissue with scar tissue, leading to progressive liver dysfunction. This condition often results from a long‐term damage secondary to hepatitis, alcohol abuse, nonalcoholic fatty liver disease, and many others. Complications of cirrhosis include portal hypertension, variceal bleeding, hepatorenal syndrome (HRS), and ascites, necessitating complex medical management to prevent severe outcomes.

In managing the complications of cirrhosis, terlipressin is often used in acute esophageal variceal bleeding (EVB) and HRS. Terlipressin is a synthetic vasopressin primarily affecting vasopressin type I (V1) receptors. It has a vasoconstrictor effect on the splanchnic vasculature that reduces the portal circulation and increases the systemic blood circulation and the effective mean arterial blood pressure, making it a beneficial vasoactive agent in conditions related to portal hypertension like EVB and HRS.^1^, ^2^ Despite its beneficial uses in patients who have complications related to liver cirrhosis, though rare, terlipressin can be the culprit for a serious ischemic complication involving the peripheral vasculature, particularly skin vasculature.^3^ Skin necrosis has been reported in rare case reports, and theories have been postulated to explore the possible mechanisms behind this drug's adverse effect.^4^, ^5^ Herein, we present a 50‐year‐old man with a history of cirrhosis complicated by acute EVB, who subsequently developed an ischemic skin lesion following terlipressin administration. Additionally, we provide a comprehensive literature review of the topic.

## 2 CASE PRESENTATION

### 2.1 Case history and examination

A 50‐year‐old man presented with two episodes of bloody vomiting and four episodes of black stools over the past 2 days. His past medical history is significant for type 2 diabetes mellitus on oral anti‐hyperglycemic therapy and hepatitis B‐related cirrhosis, which was diagnosed 10 months ago with a Child‐Pugh score of 9 (Class B) and Model for End‐Stage Liver Disease‐sodium (MELD‐Na) score of 15. He also has a history of EVB 6 months before presentation, which was managed with endoscopic banding with evidence of esophageal variceal resolution on repeated endoscopy 2 months after banding. At that time, the patient received six intravenous (IV) terlipressin boluses of 2 g every 6 h with no complications observed. The patient is not aware of any drug or food allergy.

On examination, initial vital signs were normal, with a pulse rate of 91 beats per minute and a blood pressure of 123/80 mmHg. Digital rectal examination revealed melena; otherwise, physical examinations were unremarkable.

### 2.2 Diagnosis, investigation, and treatment

Laboratory testing revealed a hemoglobin (Hb) level drop from a baseline of 13.9–8.5 gm/dl (normal range 13–17 gm/dl). The platelet count was 107 × 10^3^/uL (normal range 150–410 × 10^3^/uL), and the INR was 1.5. Liver function tests revealed total bilirubin of 34 umol/L (normal range 0–21 umol/L) with direct bilirubin of 17 umol/L (normal range 0–5 umol/L), and normal alanine aminotransferase (ALT), aspartate aminotransferase (AST), and Alkaline phosphatase (ALP).

**FIGURE 1:**
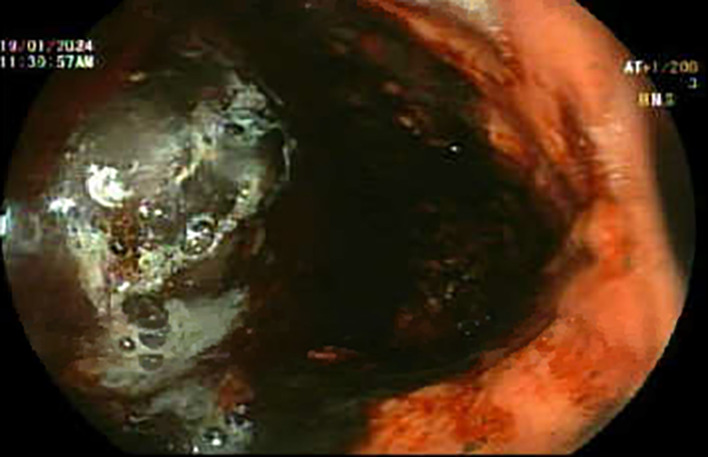
Endoscopic view showing the stomach full of blood and clots.

**FIGURE 2:**
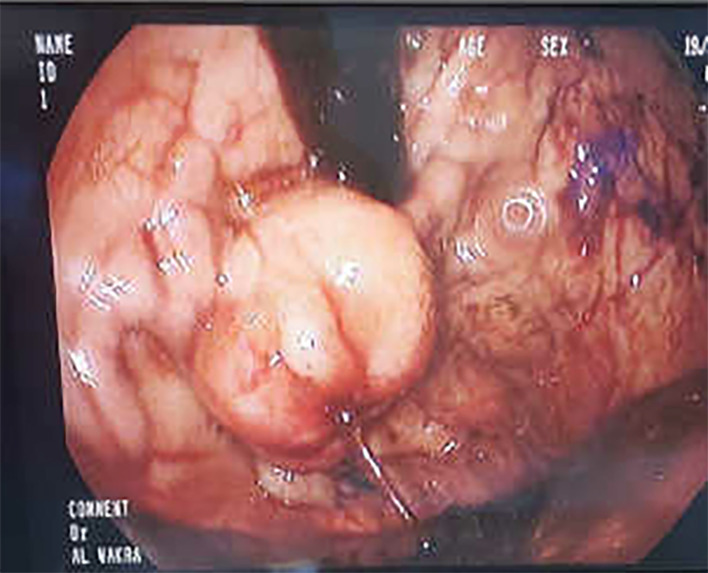
Repeated endoscopy revealing severe active bleeding from gastroesophageal varix (GOV) type 2.

The patient was resuscitated with IV fluids and started on intravenous esomeprazole and terlipressin with a dose of 2 g every 6 hours. Esophagogastroduodenoscopy (EGD) revealed stomach full of blood and clots (Figure 1), prompting procedure termination and immediate transfer to the medical intensive care unit (MICU). Subsequently, the patient was intubated, and an urgent bedside EGD was performed revealing severe active bleeding from gastroesophageal varix (GOV) type 2 (Figure 2). Endoscopic hemostasis was achieved after injecting the bleeding gastric varices with 3.5 mL Histoacryl mixed with 3.5 mL lipiodol. After that, the patient remained clinically stable, with a stable Hb level and no evidence of further melena. He was kept on intravenous esomeprazole and terlipressin.

### 2.3 Outcome and follow up

**FIGURE 3:**
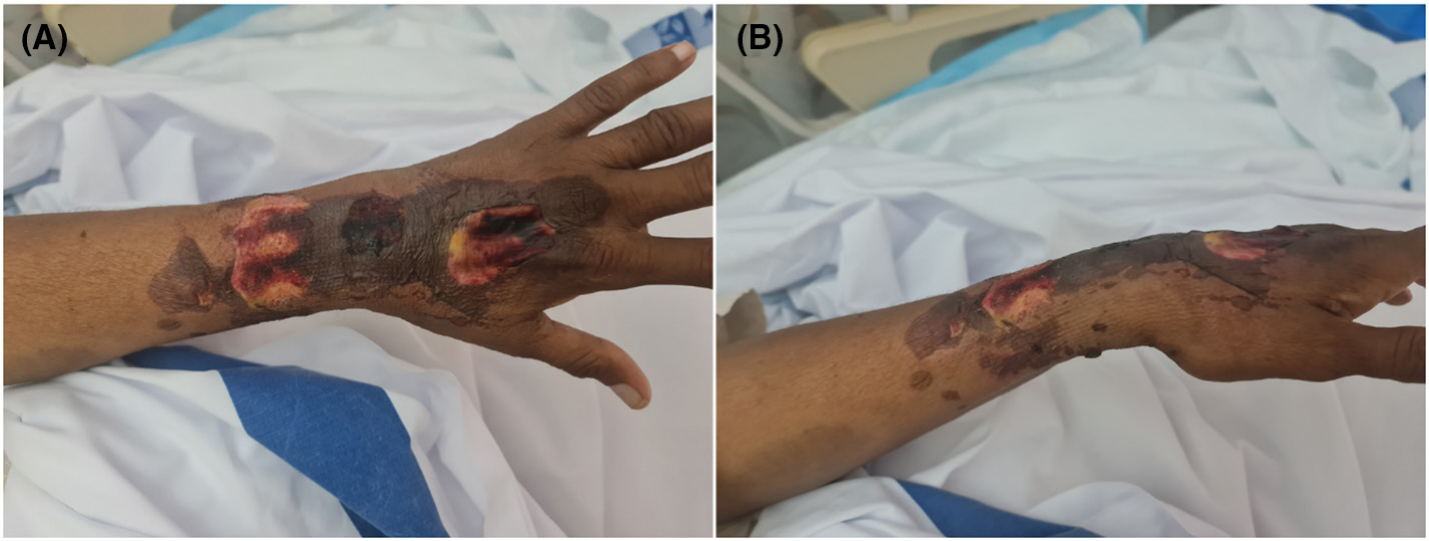
Anterior (A) and lateral (B) aspect of the dorsum of the left forearm and hand showing swelling and necrotic tissue of dark red color with ruptured bullae.

Following the administration of the ninth terlipressin dose over a period of approximately 60 hours, the patient developed left arm swelling and tightness that rapidly evolved to blistering and reddish‐black discoloration on the dorsal aspect of his left forearm and hand, accompanied by a spike of fever with a peak temperature reading of 38.4 C (Figure 3). Venous Doppler ultrasound of the left upper limb showed patent veins with normal flow. The patient refused a skin biopsy. A provisional diagnosis of terlipressin‐induced skin necrosis with possible underlying infection was made. Terlipressin was held, and the patient was started on clindamycin and piperacillin/tazobactam after a discussion with dermatology and infectious diseases teams. The skin lesions improved over the following 5 days, and he was discharged in stable condition. The patient was seen in the clinic 3 weeks after discharge. The skin lesion has almost completely healed, with no new blisters or discharges observed.

## 3 DISCUSSION

Terlipressin, also known as tricycl‐lysine vasopressin, is a synthetic analog of vasopressin. It is commonly used to treat EVB and HRS. Despite its validated benefit, terlipressin has been rarely reported to cause serious skin ischemic complications ranging from cyanosis to gangrene.^6^, ^7^ In the present study, we present a case of a 50‐year‐old gentleman with a history of CHILD‐Pugh B liver cirrhosis who developed terlipressin‐induced skin necrosis. Additionally, we provide a comprehensive literature review of case reports involving cirrhotic patients experiencing similar adverse effects.

As shown in Table 1, we analyzed a total of 18 case reports/case series of terlipressin‐induced skin necrosis comprising a total of 22 patients. Among these individuals, 78% were male, with a mean age of 51. The predominant underlying cause of liver cirrhosis was ethanol consumption in 63% of the cases, followed by viral etiology in 22.7%, nonalcoholic fatty liver disease in 13.6%, and other miscellaneous factors contributing to elevated portal hypertension. These findings parallel our case, involving a 50‐year‐old male who presented with variceal bleeding attributed to liver cirrhosis secondary to Hepatitis B infection. Among the 22 cirrhosis patients examined across the 18 case reports, the primary indications for terlipressin use in cirrhotic patients were HRS in 72.7%, followed by EVB in 31.8%.

**TABLE 1:** Clinical characteristics of the included case reports.

Author	Cirrhosis Etiology	Gender	Age	Indication	Terlipressin Dose	Onset after initiation	Location/description of the lesions
Ozel Coskun, B.D., et al.^1^	NAFLD	M	65	EVB	1 mg q4h	2 days	Erythema/Bruising/Swelling on extensor side of right forearm and hand.
Ahmed, R. et al.^2^	HCV	F	50	HRS	1 mg q6h (+ 2 mg initial bolus)	2 days	Extensive cyanosis of right hand extending to elbow. Distal phalanges of left hand and toes of both feet had a similar appearance. small bullae in right forearm.
Sudan, S., et al.^5^	Alcoholism	M	48	HRS	2 mg q4h	2 days	Reddish‐black discoloration of bilateral lower limbs.
Megarbane, H., et al.^8^	Alcoholism (+HCC)	M	68	HRS	1 mg q6h	3 days	Diffuse purpuric and necrotic plaques all over the body, including tongue and scrotum.
	Metastatic adenocarcinoma to the liver	M	74	Pseudo‐HRS	0.5 mg hourly (continuous infusion)	4 days	Isolated large erythematous and purpuric macular plaque on the scalp near metastatic skin nodules.
Donnellan, F., et al.^10^	Autoimmune liver disease	M	47	HRS	0.5 mg q6h	2 days	Bullous hemorrhagic lesions, starting in legs extending to thighs and feet.
	NAFLD	F	53	HRS	0.5 mg q4h	5 days	Extensive bruising and large exudative blistering of the skin of the abdominal wall and upper thighs.
	Alcoholism, NAFLD	M	56	EVB, HRS	1 mg q6h	3 days	Ecchymosis and blistering on the skin of the right groin and flank.
Iglesias Julián, E., et al.^13^	Alcoholism, HCV	F	84	EVB	1.5 mg q4h	2 days	Hematoma & bullous lesions over the hypogastrium and blackish‐brown, reticular shaped lesions in the flanks and periareolar regions.
Carvalho, E.B.J., et al.^14^	Alcoholism	M	72	HRS	1 mg q6hr, then increased to 2 mg q6h	1 day (after escalation of the dose)	Skin necrosis on the tip of the first digit of the left foot and cyanosis of all fingers of right foot with initial necrotic signs in 3rd‐5th digits.
Lu, Y.Y. et al.^15^	HCV	F	75	EVB	1 mg q6h	1 day	Reticulated purpuric patches appeared on the abdomen, buttocks, thighs, spread with blistering and skin detachment the next day.
Zimmer, V. et al.^16^	Alcoholism	M	54	HRS	1 mg q6h	2 days	Ischemic skin changes and necrosis in flanks and limbs and edematous scrotal skin.
Kucukdemirci, O., et al.^17^	HBV	M	67	HRS	2 mg q6h	2 days	Ecchymotic and bullous lesions on the abdomen, back, scrotum, and extremities.
Jain, G., et al.^18^	Alcoholism	M	54	HRS	2 mg q4h	3 days	Bilateral reticulated nonblanching erythematous and purpuric macules and patches with bullae in both lower limbs.
Kulkarni, A.V., et al.^19^	Alcoholism	M	54	HRS	2 mg per day (continuous infusion)	5 days	Extensive skin necrosis on the lower limbs with a gangrenous lesion on the right knee.
	Alcoholism	M	45	HRS	2 mg per day (continuous infusion)	2 days	Cyanosis of the upper hands and diffuse ischemic purpuric lesions on the lower limbs.
Posada, C., et al.^20^	Alcoholism	M	39	HRS	0.5 mg q4h	3 days	Cyanosis and pain in the acral areas and purpuric lesions appeared on the lower legs, evolving into large necrotizing areas presenting as a livedoid pattern involving the entire lower extremities.
Khandelwal, A., et al.^21^	Alcoholism	M	67	HRS, EVB	1 mg q4h (+ 2 mg initial bolus)	4 days	Blackish discoloration of the skin of all toes of the left foot along with the distal part of the foot on both the dorsal and ventral aspects and medial two toes.
Busta Nistal, M.R. et al.^22^	Alcoholism	M	45	HRS	1 mg q4h	2 days	Purpuric skin lesions appeared on both lower extremities, scrotum, and umbilical hernia. Left lower limb (had chronic lymphedema in this leg, leading to poor outcome) was extensively necrotized leading to a supracondylar amputation.
Chang, Y.H., et al.^23^	HCV	M	45	EVB	Not mentioned	2 days	Increasing pain in his right posterior thigh. Biopsy showed muscle necrosis.
Macedo, S.S., et al.^24^	Alcoholism	M	71	EVB	(3 mg 1st day, 10 mg 2nd day, 9 mg 3rd day, 3 mg 4th day)	5 days	Extensive purpuric cutaneous lesions involving the lower and upper limbs, scrotum, and abdomen.
Chiang, C.W. et al.^25^	Alcoholism	M	65	HRS	2 mg q6h then decreased to 1 mg q6h	3 days	Cold extremities and cyanosis, resulting in purple discoloration of the affected sites, including the fingers, toes, area around the umbilical hernia, and scrotum.

Abbreviations: EVB, esophageal variceal bleeding; F, female; HBV, hepatitis B virus; HCC, hepatocellular carcinoma; HCV, hepatitis C virus; HRS, hepatorenal syndrome; M, Male; NAFLD, nonalcoholic fatty liver disease.

In our case, we employed a dosing regimen consisting of a 2 mg terlipressin bolus administered every 6 h. Among the 22 patients listed in Table 1, information regarding terlipressin dosing and route of administration was available for 21. Most of these patients, 85.7%, received terlipressin via bolus dosing, as opposed to the continuous infusion (14.3%). Although the precise dosing regimens varied across studies, a common approach involved administering boluses ranging from 1 to 2 mg every 4–6 h. The onset of ischemic skin necrosis following the initiation of terlipressin, as reported in the analyzed case studies, ranged from a minimum of 2 days to a maximum of 5 days. Our case exhibited a similar timeline, with ischemic skin necrosis emerging on the third day of treatment. The location of ischemic skin necrosis exhibited notable variability across the reported studies. In our case, the affected area was on the dorsal side of the left forearm and hand. This observation aligns with findings by Coskun et al., where necrotic tissue manifested on the extensor side of the right forearm and hand, with the fingers being spared.^1^ Conversely, Megarbane et al. documented ischemic skin necrosis in atypical regions such as the trunk, scrotum, and scalp in two reported cases.^8^

Although numerous studies have investigated different mechanisms underlying terlipressin‐induced skin necrosis, the precise mechanism that justifies the observed adverse effect is not fully understood. Developing skin necrosis following terlipressin administration in cirrhotic patients is a complex phenomenon, potentially involving various factors. Terlipressin exerts its main pharmacological effect by stimulating V1 receptors of vascular smooth muscle, primarily in areas where V1 receptors are widely distributed (abdomen and thigh).^9^ This vasoconstrictive property has been implicated in compromising tissue oxygenation due to microcirculatory failure, leading to tissue ischemia and necrosis.^9^ Additionally, the activation of endothelial cell V2 receptors releases thrombogenic factors that increase platelet aggregation, leading to impairment of the distal microcirculation due to a thrombus formation.^7^ It has also been observed that obesity augments the chance of terlipressin‐induced skin necrosis, as it stretches the skin of the abdomen and lower limbs, increasing the surface area for the microvascular blood supply.^10^ Furthermore, other predisposing factors for terlipressin‐induced skin ischemia include hypovolemia, concomitant use of other vasopressors, ischemic heart disease, and spontaneous bacterial peritonitis.^11^

Similarly, the relation between the terlipressin route of administration and the incidence of adverse effects is still unclear. Furthermore, terlipressin undergoes rapid metabolism by endopeptidases once introduced into the body, transforming into the active form known as lysine vasopressin. Compared to the short half‐life of vasopressin, which lasts only about 6 min, terlipressin has a longer half‐life of approximately 6 h. The longer half‐life of terlipressin allows its administration as intermittent boluses rather than continuous infusion in the clinical setting.^12^ However, it is proposed that cutaneous necrosis was observed less in continuous infusion than bolus infusion, which aligns with what we reported earlier: 14.3% in the former route of administration.^13^

The principal approach to terlipressin‐induced skin necrosis is to withhold terlipressin, regardless of the route of administration, to promote the restoration of blood flow to the affected area and enhance skin healing.^11^ Additionally, further management with supportive care is crucial to prevent further deterioration of the lesion, including wound care, such as applying antiseptic ointments, using dressings to protect the affected area, and keeping the area clean and dry.^5^ Understanding the mechanisms driving terlipressin‐induced skin necrosis is crucial for optimizing treatment strategies and mitigating adverse outcomes in cirrhotic patients. Therefore, further research into the specific pathways leading to this complication is warranted to enhance clinical management and improve patient outcomes.

## 4 CONCLUSION

The occurrence of terlipressin‐induced skin necrosis in cirrhotic patients is a rare but serious adverse event that warrants further investigation. While the exact mechanism underlying this phenomenon remains unclear, several hypotheses have been proposed, including the potential role of vasoconstriction and impaired local circulation. Clinicians should be aware of this potential complication in cirrhotic patients receiving terlipressin therapy and closely monitor for any signs of skin necrosis. Early recognition and prompt intervention are crucial in preventing further complications and improving patient outcomes. Further research is needed to better understand the risk factors associated with terlipressin‐induced skin necrosis and to develop effective preventive strategies. Overall, healthcare providers should exercise caution when prescribing terlipressin to cirrhotic patients, weighing the potential benefits against the risks of this rare but significant adverse event.

## AUTHOR CONTRIBUTIONS

**Ashraf I. Ahmed:** Conceptualization; formal analysis; methodology; validation; visualization; writing – original draft; writing – review and editing. **Muhammad Zain Kaleem:** Conceptualization; formal analysis; methodology; validation; visualization; writing – original draft; writing – review and editing. **Shahem Abbarh:** Conceptualization; investigation; methodology; validation; visualization; writing – original draft; writing – review and editing. **Haider Hussein Barjas:** Conceptualization; validation; visualization; writing – original draft; writing – review and editing. **Abdellatif Ismail:** Conceptualization; validation; visualization; writing – original draft; writing – review and editing. **Mhd Kutaiba Albuni:** Conceptualization; methodology; visualization; writing – original draft; writing – review and editing. **Bisher Sawaf:** Conceptualization; data curation; supervision; validation; visualization; writing – original draft; writing – review and editing.

## FUNDING INFORMATION

This research did not receive any specific grant from funding agencies in the public, commercial, or not‐for‐profit sectors.

## CONFLICT OF INTEREST STATEMENT

The authors report no conflict of interest.

## ETHICS STATEMENT

The article was approved by the Institution Review Board at Hamad Medical Corporation.

## CONSENT

A written informed consent was obtained from the patient for the publication of all images, clinical data and other data included in the manuscript. All identifying information has been removed.

## Data Availability

The data that support the findings of this study are available from the corresponding author uponreasonable request.
